# Spatial dataset on future forest cover changes in Southeast Asia projected under the shared socioeconomic pathways

**DOI:** 10.1016/j.dib.2023.109705

**Published:** 2023-10-20

**Authors:** Ronald C. Estoque

**Affiliations:** Center for Biodiversity and Climate Change, Forestry and Forest Products Research Institute, 1 Matsunosato, Tsukuba, Ibaraki 305-8687 Japan

**Keywords:** Forest monitoring, Forest change projection, Land change, Climate change, Biodiversity, Tropical region

## Abstract

The five shared socioeconomic pathways (SSPs) provide future projections for various social-ecological variables, including forest cover. However, these projections are limited to quantities and lack a spatial dimension. This dataset is the result of an effort to spatialize the projected forest cover changes (losses and gains) (2015-2050) in Southeast Asia under the five baseline SSPs. The dataset is available in GIS raster format (.tif) at a spatial resolution of 300 m. A wide range of users can benefit from this dataset, as it can be used independently or in conjunction with other datasets. Specifically, it can be employed to assess potential future social-ecological impacts, both positive and negative, in Southeast Asia resulting from changes in forest cover. The dataset supports analysis at the national, sub-national, or landscape levels.

Specifications Table

**Table:** 

Subject	Environmental Science, Earth and Planetary Sciences
Specific subject area	Cutting across the areas of land change science (forest cover change), geoenvironmental science, climate science, and nature and landscape conservation
Data format	GIS raster format (.tif)
Type of data	Geospatial data
Data collection	This dataset was produced using a spatially explicit land change modeling procedure focusing on potential future forest cover changes under the five baseline shared socioeconomic pathways (SSPs) [1,2]. The five SSPs are: SSP1, or the sustainability/taking the green road scenario; SSP2, or the middle of the road scenario; SSP3, or the regional rivalry/rocky road scenario; SSP4, or the inequality/road divided scenario; and SSP5, or the fossil-fueled development/taking the highway scenario [3,4]. The land change modeling procedure involved three primary steps, namely change quantification, transition potential modeling, and spatial allocation of the quantified projected change, which can either be forest loss or forest gain depending on the pathway.
Data source location	Forestry and Forest Products Research Institute, Tsukuba City, Japan
Data accessibility	Repository name: Zenodo Data identification number: 10.5281/zenodo.8162177 Direct URL to data: https://doi.org/10.5281/zenodo.8162177 Instructions for accessing these data: N/A
Related research article	R.C. Estoque, M. Ooba, V. Avitabile, Y. Hijioka, R. Dasgupta, T. Togawa, Y. Murayama, The future of Southeast Asia's forests, Nat Commun 10, 1829 (2019). https://doi.org/10.1038/s41467-019-09646-4

## 1 Value of the Data

• The five SSPs provide future projections on various social-ecological variables, including forest cover. However, these projections are limited to quantities and lack a spatial dimension. In this context, the current dataset holds value as it presents downscaled or spatialized projections of future forest cover changes specifically in Southeast Asia.
• A wide range of users can benefit from this dataset, particularly those involved in monitoring and projecting forest cover changes, as well as in downscaling the projected land use changes under the five SSPs, including forest cover changes.
• Other researchers can utilize this dataset either independently or in conjunction with other datasets. Specifically, it can be employed to assess potential future social-ecological impacts, both positive and negative, in Southeast Asia resulting from changes in forest cover (both loss and gain). The dataset supports analysis at the national, sub-national, or landscape levels.

## 2 Data Description

**Fig. 1:**
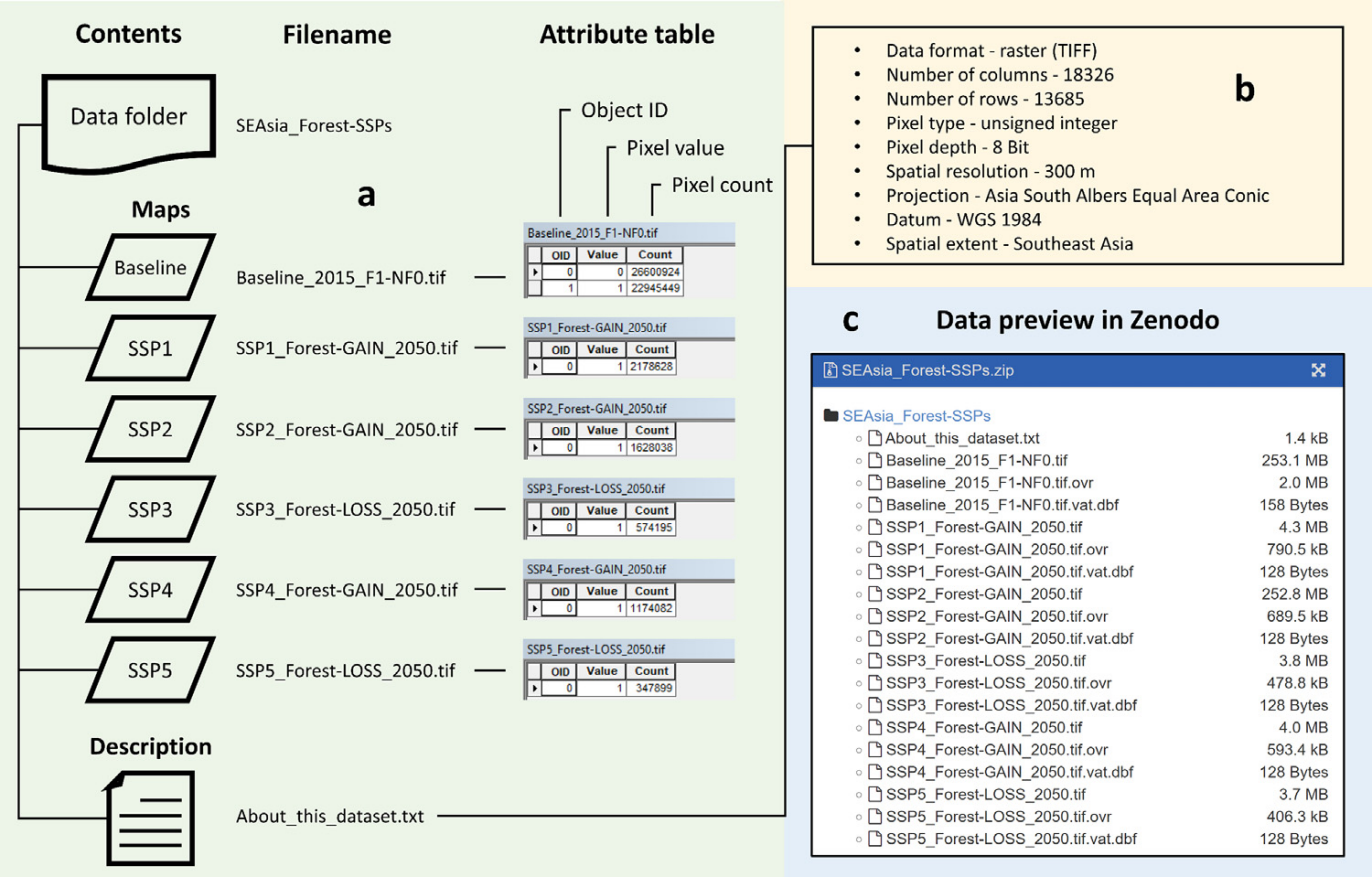
Data structure. (a) Contents of the data folder; (b) some information contained in the txt file; and (c) preview screen of the dataset in the download page of Zenodo.

The entire dataset includes six maps enclosed in a data folder (Fig. 1a) [2]: (1) 2015 baseline forest (F) and non-forest (NF) map, with pixel values of 1 (F1) and 0 (NF0), respectively; (2) SSP1 2050 projected net forest gain map; (3) SSP2 2050 projected net forest gain map; (4) SSP3 2050 projected net forest loss map; (5) SSP4 2050 projected net forest gain map; and (6) SSP5 2050 projected net forest loss map. Each map layer has an attribute table with three columns, namely OID (object ID), Value (pixel value or class code), and Count (pixel count). Unlike the baseline map with two classes (F and NF) as mentioned above, the SSP maps contained only one class with a value or class code of 1 which represents either forest loss or forest gain depending on the SSP.

Additionally, a txt file describing the dataset is also included in the data folder (Fig. 1a and b). The maps are in GIS raster format (.tif) projected to the Asia South Albers Equal Area Conic projection system and the WGS 1984 datum. They have a spatial resolution of 300 m and a number of columns and rows of 18,326 and 13,685, respectively. The pixel type is unsigned integer and the pixel depth is 8 Bit. The spatial extent of the dataset covers the entire Southeast Asia.

As shown in the data preview of Zenodo's download page, each map layer comes with two other files (.ovr and .dbf) (Fig. 1c). These files are necessary to display the attribute table of each map layer (e.g. in ArcGIS). In the absence of these files, the attribute table of each map layer can be created. In addition to ArcGIS, the maps (.tif) can also be opened, read and processed in other GIS software packages. For example, the maps can also be opened, read and processed directly in QGIS, or indirectly in TerrSet in which the maps first need to be exported/imported to the desired format (.rst). Furthermore, the maps can be opened, read and processed using R and other programming languages such as Python.

## 3 Experimental Design, Materials and Methods

**Fig. 2:**
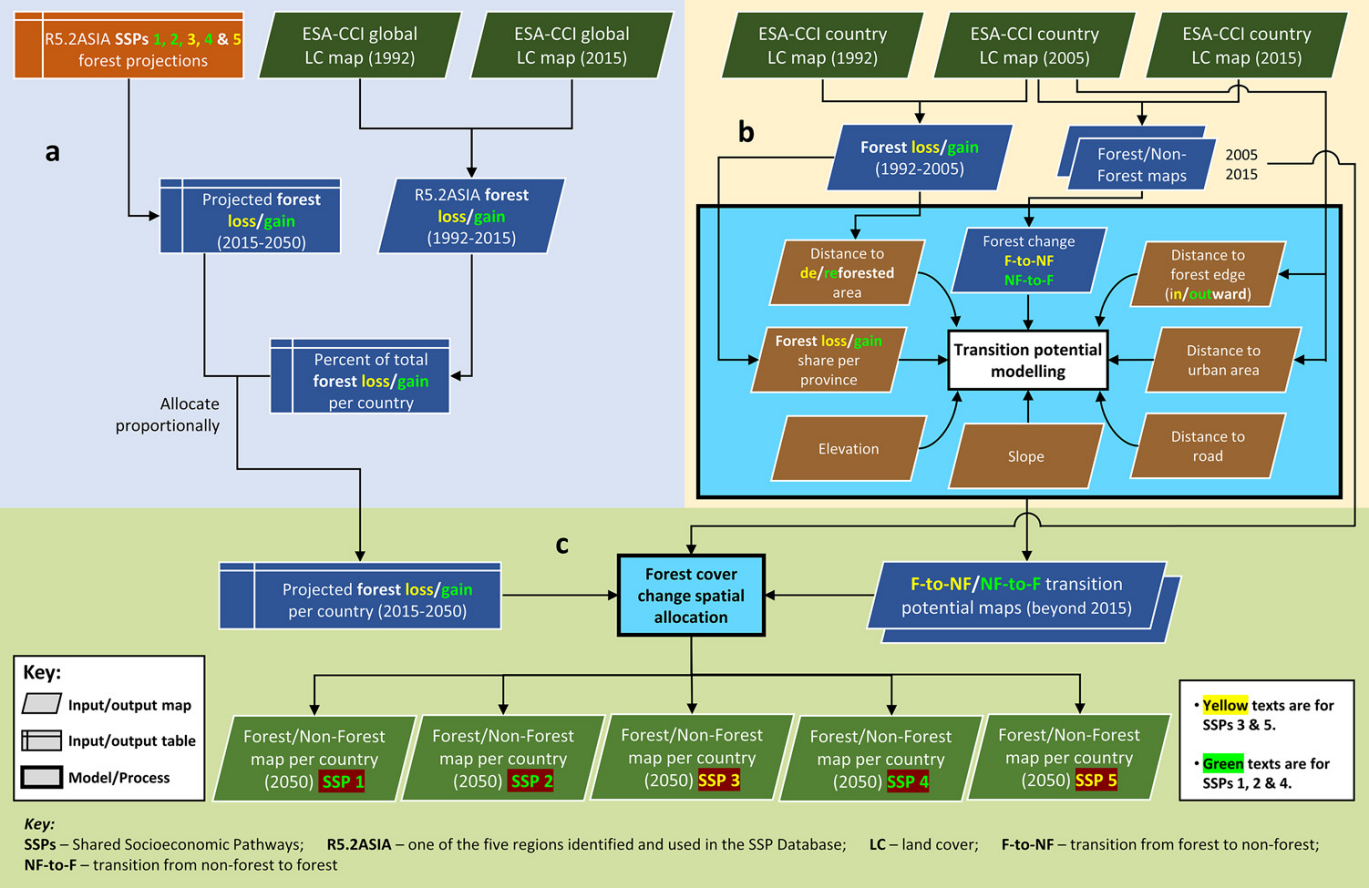
Flowchart of the spatially explicit land change modeling procedure. This procedure was developed and used to spatially allocate the projected quantities of forest cover changes under the five baseline SSPs. (a) Forest cover change quantification, (b) transition potential modeling, and (c) forest cover change spatial allocation. Source: Estoque et al. [1]

To provide a spatial dimension to the projected future forest cover changes under the five SSPs, a spatially explicit land change modeling procedure was employed. This modeling procedure (Fig. 2) involved three primary steps, namely change quantification, transition potential modeling, and spatial allocation of the quantified projected change [1]. Here, land change specifically refers to forest cover change (loss and gain).

From 2015 to 2050, the forest cover in Asia (one of the five SSP regions) would increase under SSPs 1, 2 and 4, but would decrease under SSPs 3 and 5 (SSP Public Database, Version 1.1). For the subsequent data processing, the GIS administrative country boundary data (vector polygon) [5], along with the member countries of the Asian region identified in the SSP Public Database, was used to spatially delineate the extent of the Asian region as per the SSP definition. The resulting GIS country boundary map of the Asian region was projected to the Albers projection system.

To quantify the future forest cover changes in each country under the five SSPs (Fig. 2a), first, the Asian region's overall future forest cover change between 2015 and 2050 under each SSP was quantified using the SSP Public Database. To achieve this, for each SSP, the 2015 forest area was subtracted from the 2050 forest area (forest gain for a positive difference and forest loss for a negative difference).

Second, the percentage share of each country to the Asian region's past forest cover loss and gain was quantified. To accomplish this, the ESA-CCI land cover maps in 1992 and 2015 (v2.07) [6] were used. These time points were the earliest and latest available time points at the time of data processing. These maps have a spatial resolution of 300 m and were also projected to the same Albers projection system using the nearest neighbor resampling technique. Their land cover classes were reclassified into two classes, namely forest (F) (class codes: 50, 60-62, 70-72, 80-82, 90, 160, 170) and non-forest (NF) (other classes) [7]. Subsequently, the resulting 1992 and 2015 F/NF maps were cross-tabulated (combined) to determine the past gross forest loss and gain in the region. A F/NF map for 2005 was also produced for the subsequent data processing procedures.

And third, a zonal analysis was performed to quantify the extent of past gross forest loss and gain (1992-2015) within each country. For this purpose, the countries were used as distinct zones, employing the GIS country boundary map, while the outcome of the previous cross-tabulation step served as the input raster. Subsequently, the proportion or percentage share of each country's contribution to the past total forest cover loss and gain in the Asian region was computed. These individual country percentages were then utilized as a multiplicative factor to proportionally allocate across all countries within the Asian region the projected forest cover losses and gains (2015–2050) under the five SSPs. The derived data for the Southeast Asian countries were used in the subsequent data processing.

For the transition potential modeling (one for forest loss and one for forest gain), spatial variables were used (Fig. 2b). For forest loss, the distinct spatial variables used in transition potential modeling were ‘distance to deforested area (1992-2005)’, ‘2005 distance to forest edge - inward’, and ‘forest loss share per province (1992-2005)’. For forest gain, the distinct spatial variables were ‘distance to reforested area (1992-2005)’, ‘2005 distance to forest edge - outward’, and ‘forest gain share per province (1992-2005)’. The common spatial variables used were ‘2005 distance to urban area’, ‘distance to road’, ‘elevation’, and ‘slope’. Altogether, seven variables were used in transition potential modeling for forest loss and seven also for forest gain. Here, distance refers to Euclidean distance for all the distance variable maps.

The ‘elevation map’ was derived from the 30-m Shuttle Radar Topography Mission (SRTM) data [8]. To be spatially consistent with the F/NF maps, it was resampled to 300 m using the bilinear resampling technique. The ‘slope map’ was derived from this resampled elevation map. The ‘distance to road map’, which was also set to 300 m spatial resolution, was produced using the road network data available [9]. The ‘distance to urban map’ was produced using the urban class (class code: 190) in the 2005 ESA-CCI land cover map. To derive the ‘forest loss/gain share per province (1992-2005)’, the same zonal analysis procedure explained above was performed, but this time using the provinces as distinct zones and the outcome of the cross-tabulation between the 1992 and 2005 F/NF maps as the input raster. The GIS provincial boundary layer was also sourced from [5] (regency level for Indonesia and district level for Myanmar and Malaysia). All the spatial data were projected onto the Albers projection system to be consistent with the F/NF maps.

The purpose of the transition potential modeling was to determine which pixels had the higher likelihood to gain (under SSPs 1, 2 and 4) and lose (under SSPs 3 and 5) forest. In order to accomplish this, the Land Change Modeler's (LCM) Multi-Layer Perceptron Neural Network algorithm [10] inside the TerrSet (a geospatial monitoring and modeling software) [11] was used. Transition potential modeling was conducted for each Southeast Asian country. Further details on how the algorithm works are given in [1].

Finally, for the spatial allocation of the projected forest cover change, the quantified forest cover changes and the modeled transition potential maps were used as inputs (Fig. 2c). This step also employed the LCM and further details about this step are also given in [1]. The spatial allocation procedure was performed for each Southeast Asian country. The resulting simulated 2050 F/NF maps for each country were subsequently cross-tabulated (combined) with the 2015 F/NF map to detect and extract the spatially allocated projected forest cover gains under SSPs 1, 2 and 4, and losses under SSPs 3 and 5. Lastly, the extracted spatially allocated projected forest cover gains or losses in the 11 Southeast Asian countries were mosaicked, resulting into five regional (Southeast Asia) projected forest cover change maps (gains for SSPs 1, 2 and 4, and losses for SSPs 3 and 5).

## Limitations

As in the Food and Agriculture Organization's Global Forest Resources Assessment reports, specifically on forest extent at several time points from the recent past to present [12], the SSPs’ projected extents of land use classes across time points into the near future, including those of forest, refer to values that would result in a net change when the difference between two projected values at two different time points is determined. It would have been useful if the extent of gross losses and gains in the SSPs’ land use classes had also been projected and reported. This could have allowed the simultaneous simulation of gross forest loss and gain under each SSP. Consequently, in this dataset, the spatially allocated forest cover changes under the five baseline SSPs refer to net changes (net forest loss or net forest gain depending on the SSP).

## Ethics Statement

The author has read and followed the ethical requirements for publication in Data in Brief and confirming that the current work does not involve human subjects, animal experiments, or any data collected from social media platforms.

## CRediT Author Statement

For this Data Article: **Ronald C. Estoque –** Conceptualization and writing. For the Dataset and the Original Article: **Ronald C. Estoque, Makoto Ooba, Valerio Avitabile, Yasuaki Hijioka, Rajarshi DasGupta, Takuya Togawa, and Yuji Murayama** – Co-authors.

## Data Availability

Simulated spatially explicit dataset (300 m) on future forest cover changes in Southeast Asia projected under the baseline shared socioeconomic pathways (Original data) (Zenodo)
